# Supplementary material for: Enterobactin and salmochelin S4 inhibit the growth of Staphylococcus aureus
Source: Front Cell Infect Microbiol. 2025 Mar 5;15:1456046. doi: 10.3389/fcimb.2025.1456046 (PMC11919883; doi:10.3389/fcimb.2025.1456046)
Supplement: Supplementary file 1 [file DataSheet1.pdf]

Supplementary Figures

Figure 1S. The effect of combinations of salmochelin S4 or enterobactin with lincomycin (A) and salmochelin S4 with linezolid (B) on the growth *S. aureus* strain ATCC 25923. Sal - salmochelin, Ent - enterobactin, Linco – lincomycin, Lin – linezolid, Growth control - without siderophore and antibiotic.

A.

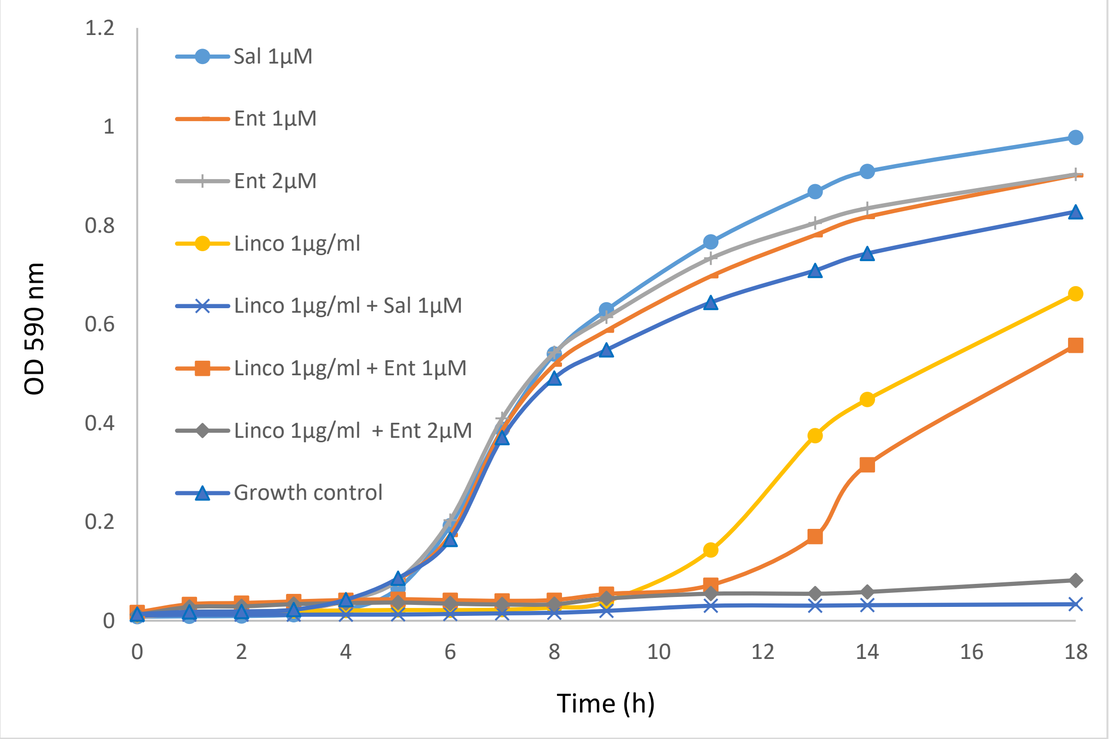

B.

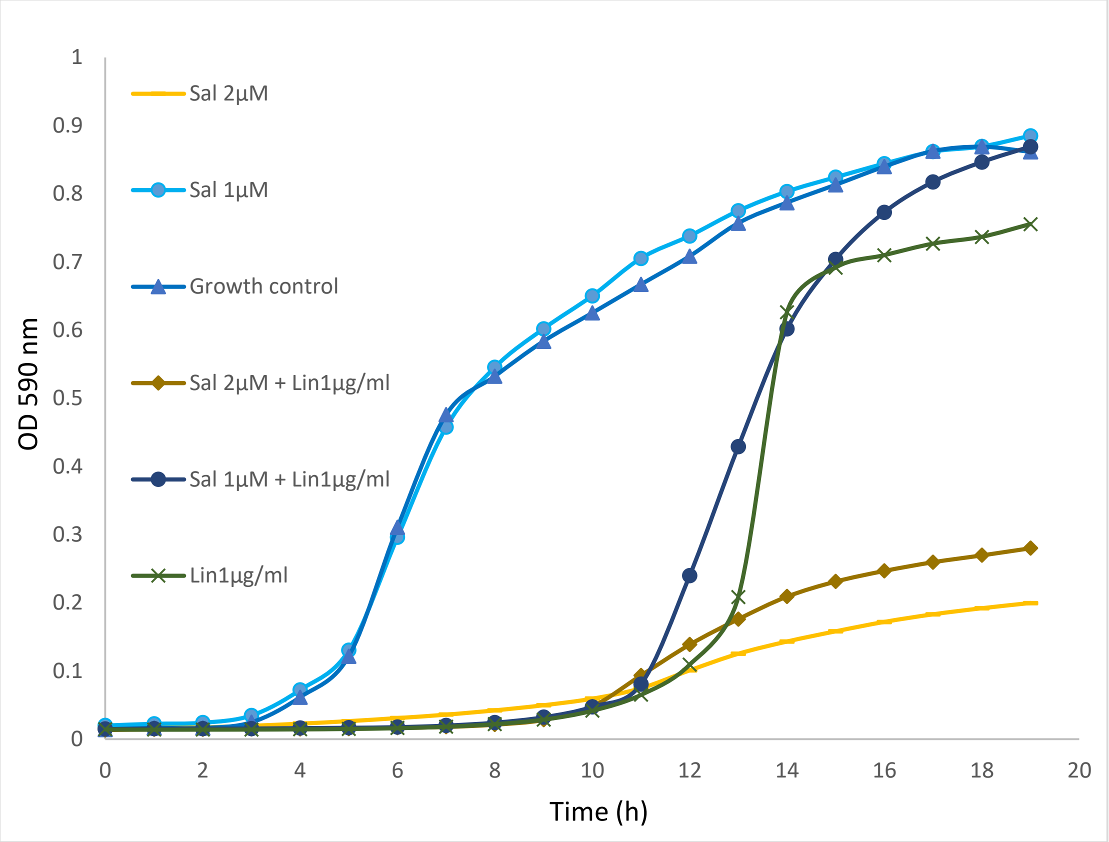

Figure 2S. Enterobactin (A) and salmochelin S4 (B) inhibits the growth of VRE clinical strain 195 in a dose- dependent manner. Sal - salmochelin, Ent - enterobactin, Growth control - without siderophore. Similar results were observed in other VRE clinical strains.

A.

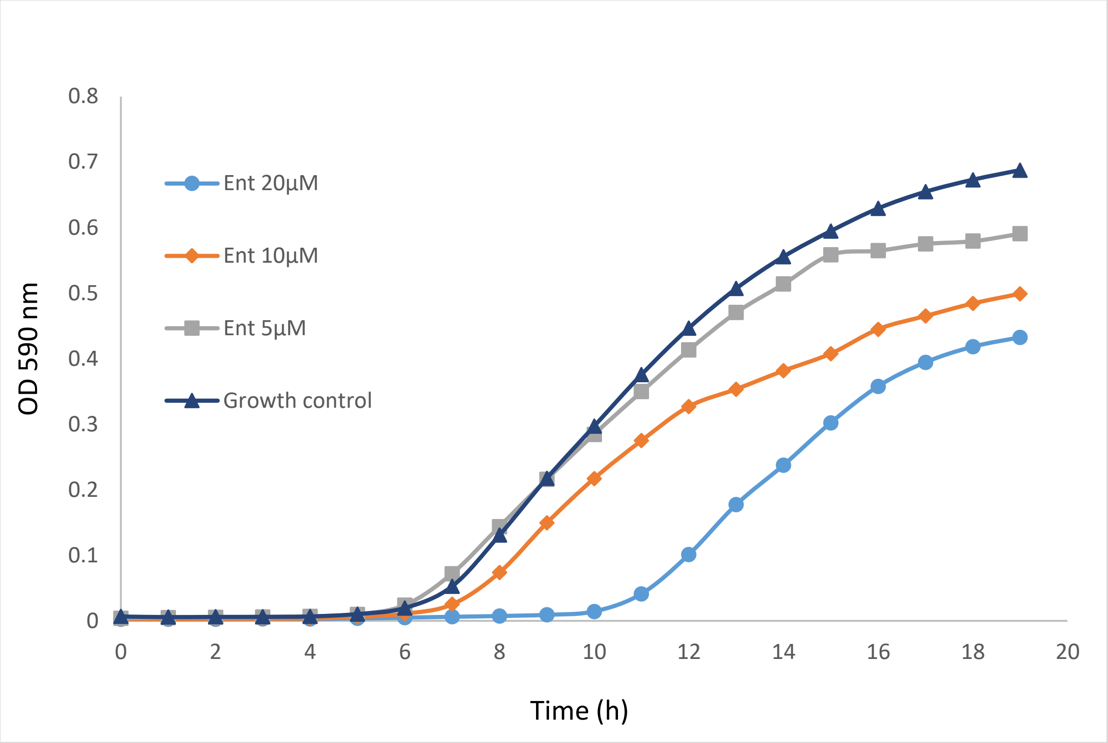

B.

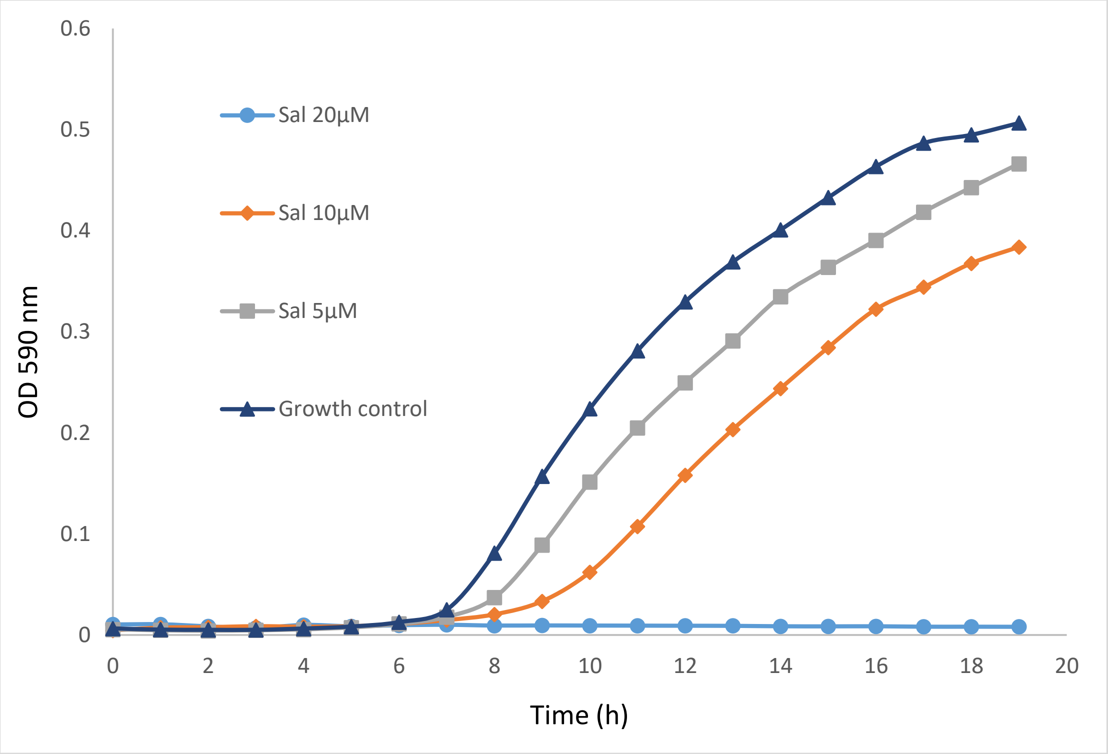

Figure 3S. The inhibitory activity of enterobactin and salmochelin S4 on Gram-negative bacteria. (A) *Klebsiella pneumonia* carbapenemase clinical strain (B)

Acinetobacter baumannii clinical strain (C) *Pseudomonas aeruginosa* ATCC 9027 (D) Uropathogenic *Escherichia coli* CFT073. Sal - salmochelin, Ent – enterobactin, Growth control - without siderophore.

A.

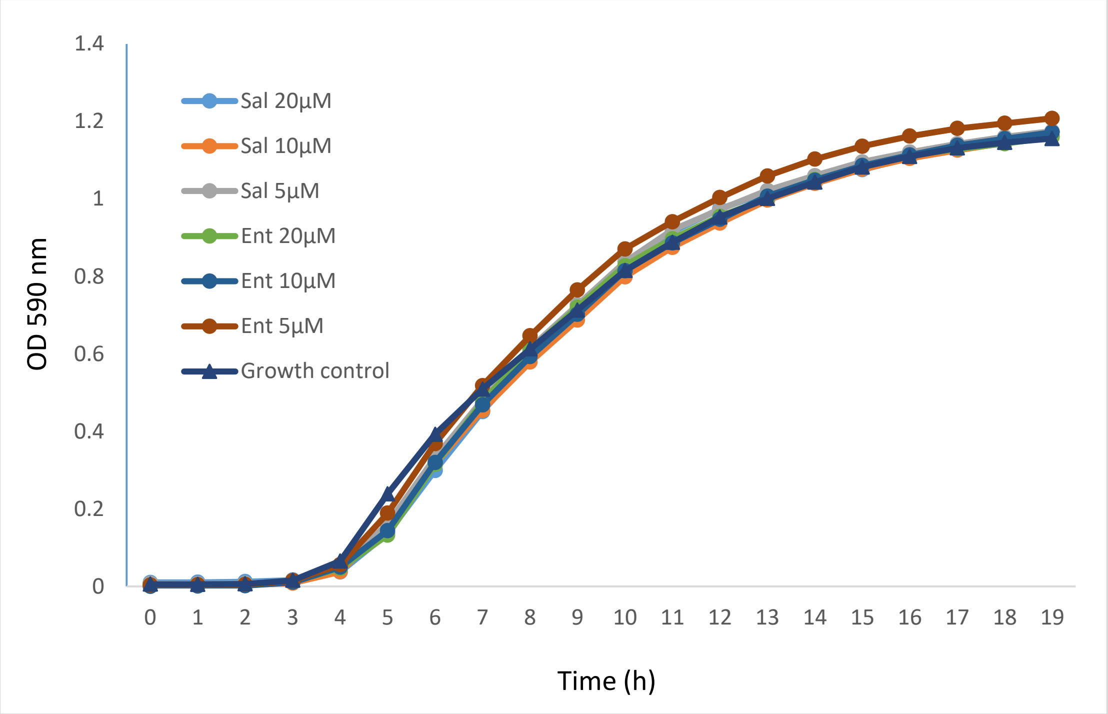

B.

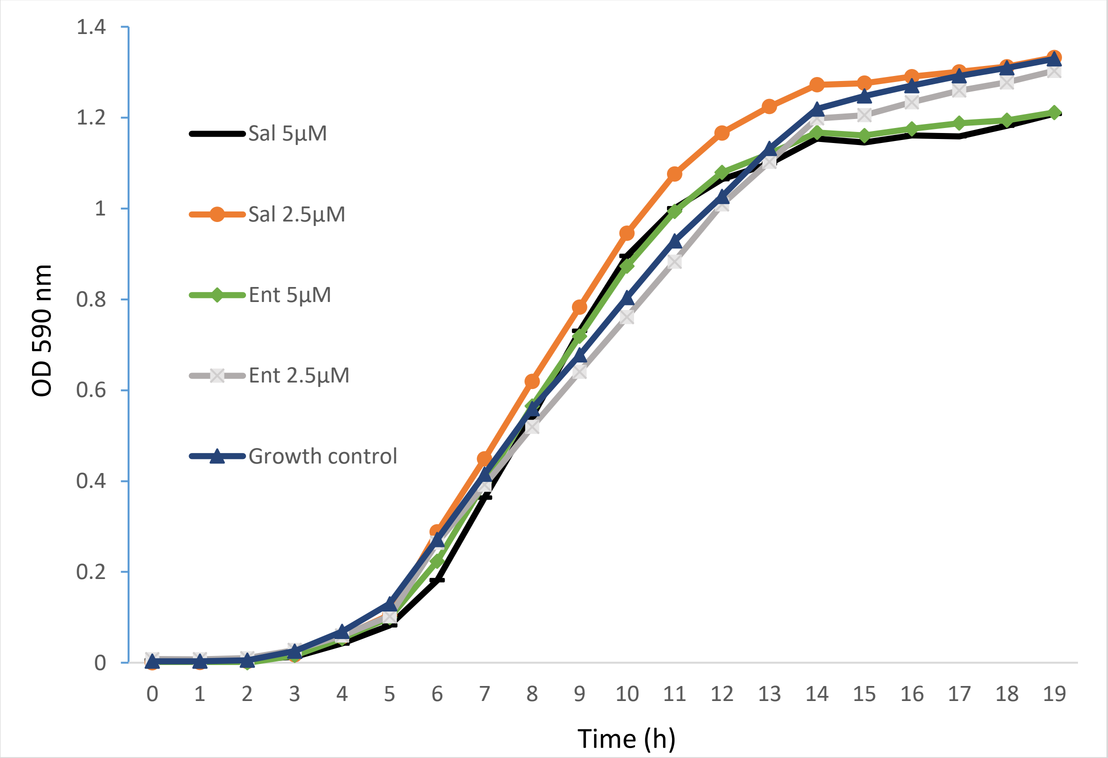

C.

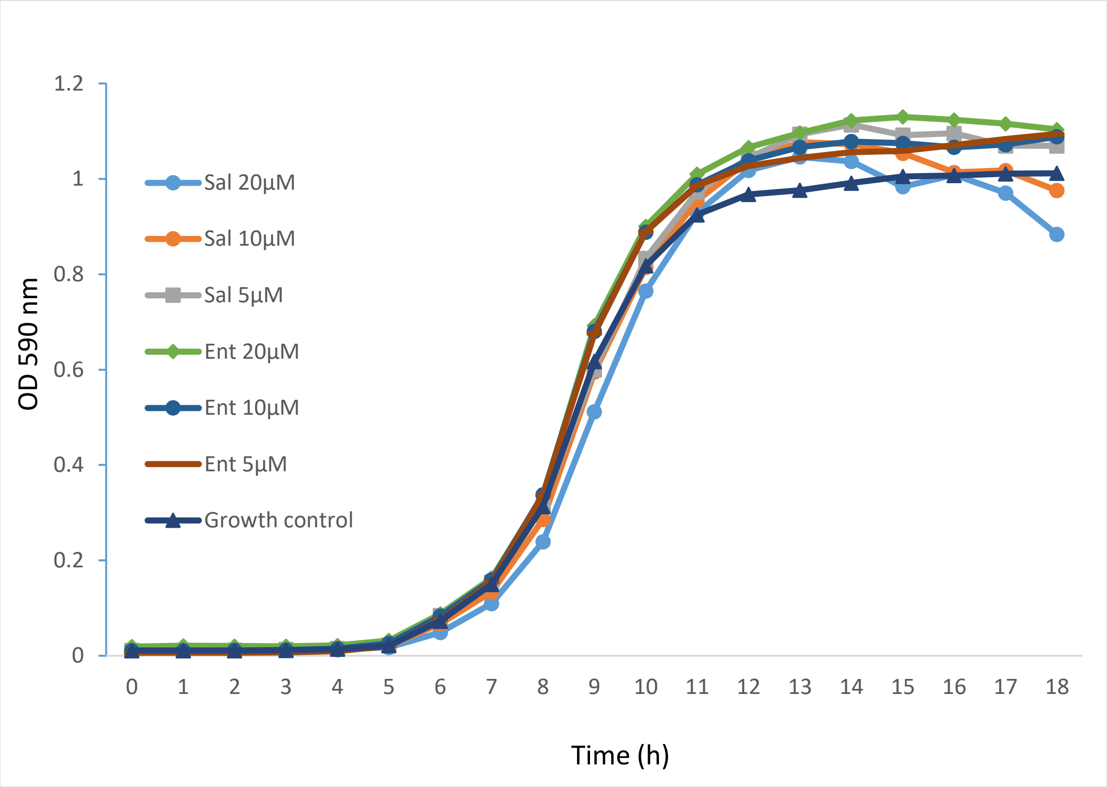

1.2
1
0.8
0.6
0.4
0.2
0
OD 590 nm
Sal 20µM
Sal 10µM
Sal 5µM
Ent 20µM
Ent 10µM
Ent 5µM
Growth control
0 1 2 3 4 5 6 7 8 9 10 11 12 13 14 15 16 17 18
Time (h)

D.

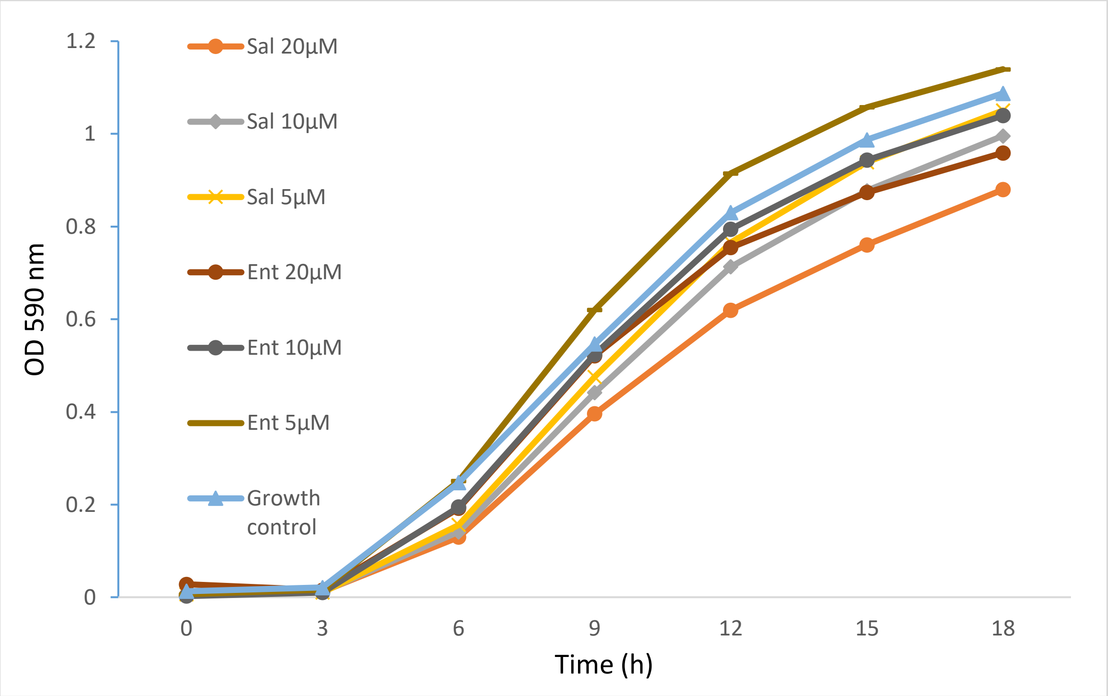

1.2
1
0.8
0.6
0.4
0.2
0
OD 590 nm
Sal 20µM
Sal 10µM
Sal 5µM
Ent 20µM
Ent 10µM
Ent 5µM
Growth control
0 3 6 9 12 15 18
Time (h)
